# Design and Initial Validation of an Infrared Beam-Break Fish Counter (‘Fish Tracker’) for Fish Passage Monitoring

**DOI:** 10.3390/s25134112

**Published:** 2025-07-01

**Authors:** Juan Francisco Fuentes-Pérez, Marina Martínez-Miguel, Ana García-Vega, Francisco Javier Bravo-Córdoba, Francisco Javier Sanz-Ronda

**Affiliations:** 1 Group of Applied Ecohydraulics, Department of Agricultural and Forest Engineering, Sustainable Forest Management Research Institute, University of Valladolid, ETSIIAA, Avenida de Madrid 44, Campus La Yutera, 34004 Palencia, Spain; ana.garcia.vega@uva.es (A.G.-V.); jsanz@uva.es (F.J.S.-R.); 2 Group of Applied Ecohydraulics, Centro Tecnológico Agrario y Agroalimentario ITAGRA.CT, Avenida de Madrid 44, Campus La Yutera, 34004 Palencia, Spain; fjbravo@itagra.com

**Keywords:** fish passage monitoring, infrared beam-break sensor, open-source hardware, non-invasive sensing, low-cost fish counter, aquatic sensor systems, fish morphometrics, adaptive river management

## Abstract

Effective monitoring of fish passage through river barriers is essential for evaluating fishway performance and supporting adaptive river management. Traditional methods are often invasive, labor-intensive, or too costly to enable widespread implementation across most fishways. Infrared (IR) beam-break counters offer a promising alternative, but their adoption has been limited by high costs and a lack of flexibility. We developed and tested a novel, low-cost infrared beam-break counter—FishTracker—based on open-source Raspberry Pi and Arduino platforms. The system detects fish passages by analyzing interruptions in an IR curtain and reconstructing fish silhouettes to estimate movement, direction, speed, and morphometrics under a wide range of turbidity conditions. It also offers remote access capabilities for easy management. Field validation involved controlled tests with dummy fish, experiments with small-bodied live specimens (bleak) under varying turbidity conditions, and verification against synchronized video of free-swimming fish (koi carp). This first version of FishTracker achieved detection rates of 95–100% under controlled conditions and approximately 70% in semi-natural conditions, comparable to commercial counters. Most errors were due to surface distortion caused by partial submersion during the experimental setup, which could be avoided by fully submerging the device. Body length estimation based on passage speed and beam-interruption duration proved consistent, aligning with published allometric models for carps. FishTracker offers a promising and affordable solution for non-invasive fish monitoring in multispecies contexts. Its design, based primarily on open technology, allows for flexible adaptation and broad deployment, particularly in locations where commercial technologies are economically unfeasible.

## 1. Introduction

River connectivity is fundamental for maintaining healthy fish populations, allowing access to critical habitats required for various life stages, including feeding, refuge, and reproduction [1]. Anthropogenic barriers such as dams and weirs fragment river systems globally, impeding these necessary movements [2]. To mitigate these impacts, engineered structures known as fish passes or fishways—which are essentially channels, lifts, or other devices (e.g., vertical-slot fishways, nature-like fishways) designed to enable fish to overcome these obstacles—are increasingly constructed [3,4]. However, the mere presence of a fishway does not guarantee effective passage for the target fish community [5]. Evaluating the biological effectiveness of these structures is therefore crucial for ensuring conservation goals are met, fulfilling regulatory requirements (e.g., the Water Framework Directive in Europe [6]), guiding adaptive management strategies, and optimizing future designs. This necessitates reliable and often long-term monitoring methodologies to accurately assess fish abundance, movement patterns, and fishway utilization.

Traditional methods for fishway assessment often involve direct capture (e.g., trapping within the fishway, netting, electrofishing) and/or marking techniques (e.g., VIE, PIT tagging, radio/acoustic telemetry) [7]. Despite providing valuable species-specific or individual-based data, these approaches have significant limitations. They are typically labor-intensive and costly, particularly for continuous or long-term monitoring programs. Furthermore, direct capture and handling are invasive methods with the potential to cause physiological stress, injury, and alterations to natural behavior, which can confound passage assessments [8,9]. However, adherence to established best-practice handling protocols aims to minimize these impacts [10]. Tagging studies, while powerful, usually only provide information on a fraction of the population and can be limited by factors such as tag life and detection efficiency. Consequently, these methods may provide only temporally discrete snapshots or biased perspectives on fish passage and population dynamics [11].

To address the limitations of traditional methods, various automated, non-contact monitoring technologies have emerged, including hydroacoustic, video, electrical resistivity, and infrared optical systems [12]. While each offers advantages, they also present challenges such as operational constraints in complex environments, sensitivity to environmental conditions like turbidity, high data-processing demands, or significant costs. Infrared beam-break counters, for instance, show promise, but their wider adoption can be limited by cost and operational limitations [13].

Given the limitations of existing commercial systems, coupled with the growing regulatory and management need for continuous monitoring to evaluate fishway functionality and support adaptive management strategies (e.g., EU Biodiversity Strategy 2030 [14]), there is a clear demand for automated monitoring solutions that meet criteria for affordability, adaptability, and reliability. This is particularly true for the vast number of existing fishways that were not originally designed to accommodate such equipment. Acquiring continuous, long-term data on fish abundance and passage dynamics is vital for effective adaptive management and for truly understanding fishway functionality across diverse conditions [15]. Addressing this well-established need and leveraging recent advances in open technology solutions and knowledge, we hypothesized that this economic barrier could be significantly lowered by developing a system based on widely accessible, low-cost, open-source hardware and software components, thereby promoting its wider application across numerous fishways and ultimately contributing to enhanced fish migration.

In this paper, we present the design, development, and initial validation of a novel low-cost fish counter based on infrared beam-break technology (hereafter ‘Fish Tracker’). The primary novelty of ‘Fish Tracker’ lies in its integration of readily available, low-cost, open-source hardware (Arduino, Raspberry Pi) and software (Python) components to create an affordable and adaptable monitoring solution. This approach allows the system to achieve core functionalities—including fish count, directionality, passage speed, and silhouette generation—at a fraction of the cost of existing commercial alternatives. We detail the specific hardware configuration, the detection and filtering algorithms implemented, and the results from laboratory and semi-natural validation trials using fish replicas and live fish (bleak and ornamental carp). The objective of this work was to develop and test this open-source-based, low-cost tool to facilitate the widespread, continuous monitoring essential for evaluating fish passage effectiveness and supporting river restoration efforts.

## 2. Materials and Methods

### 2.1. Review of Fish Monitoring Technologies and Rationale for Selection

Monitoring fish passage at river barriers is critical to evaluate fishway effectiveness and compliance with ecological restoration goals. To address the limitations of traditional methods such as manual trapping and tagging automatic fish counters offer a non-contact alternative that enables continuous, broad-scale monitoring and supports adaptive management frameworks. These systems are broadly classified as hydroacoustic, resistivity-based, mechanical, and optical (video and infrared) systems [12,16].

**Hydroacoustic systems** (e.g., DIDSON imaging sonar) detect fish by emitting ultrasound and analyzing echoes reflected mainly from the swim bladder. They operate effectively in turbid or low-light conditions and open channels, providing non-intrusive, continuous monitoring with relatively high accuracy and spatial coverage. However, their deployment in fishways can be constrained by boundary reflections, turbulence interference, limited detection range, and high data-processing demands, resulting in higher operational costs and complexity [17,18,19].

**Resistivity counters** detect fish by measuring changes in electrical conductivity as the fish passes through an electrode field [16]. These systems can reliably operate in turbid and low-visibility waters and are effective for continuous, automated monitoring of larger fish. However, they require consistent environmental conditions and regular calibration and provide limited directional or morphometric information. Additionally, installations usually require confined channels to ensure accurate detection [16,20].

**Mechanical counters** detect fish through direct physical interaction, typically triggering a flap gate or trap mechanism upon passage. These systems are economical and simple to operate, effectively handling large volumes of fish at high-density sites. Nevertheless, these systems tend to alter hydraulic conditions, obstruct fish passage, require high maintenance due to debris accumulation, and generally provide only basic passage counts without detailed biological data [7].

**Optical video systems** offer detailed visual information, enabling precise species identification and behavioral observations. When combined with machine learning, video systems can automate data processing significantly [21]. However, video performance can significantly decrease under turbid or variable lighting conditions, requires considerable data storage and processing capacity, and demands regular maintenance to prevent biofilm accumulation on camera lenses [13,22]. Additionally, artificial illumination may affect fish behavior [23].

**Infrared beam-break systems**, such as the commercial VAKI Riverwatcher, detect fish via interruptions of infrared (IR) beams [12]. These systems are effective in low-light environments, relatively resilient against moderate turbidity, and provide data on fish size, passage direction, and speed. They have fewer infrastructure and maintenance demands compared to video systems, though their performance diminishes with severe turbidity and simultaneous fish passages [13]. Despite these advantages, commercial IR counters often have high costs, partly due to specialized underwater components and limited production volumes.

A variant of IR counters includes **laser-based optical systems** and 3D laser imaging technologies. In principle, these systems can offer higher resolution and allow for narrower emitter spacing, thereby increasing detection precision. Although we found no documented field applications of these technologies in riverine environments, they have demonstrated accurate 3D reconstruction capabilities in aquaculture and experimental flume studies, enabling non-invasive biomass estimation and morphometric analysis [24]. Nevertheless, laser systems require precise alignment, are highly sensitive to biofouling, and involve significant hardware and maintenance costs. These limitations currently restrict their use to controlled or semi-controlled environments.

Considering these factors, the IR beam-break approach was selected for the Fish Tracker system. This choice was primarily driven by technology’s optimal balance between material cost-effectiveness (leveraging affordable components), operational robustness, and the sufficiency of data provided for typical management objectives. The Fish Tracker design specifically employs high-intensity infrared LEDs (Light-Emitting Diodes), facilitating straightforward alignment when compared to laser beams. Furthermore, by using open-source platforms (Arduino, Raspberry Pi), Fish Tracker substantially reduces implementation costs (material cost < 2000 EUR), making the system accessible and adaptable to varied fishway structures and conditions.

### 2.2. Basic Working Principle of the Infrared Beam-Break Counter

**Figure 1:**
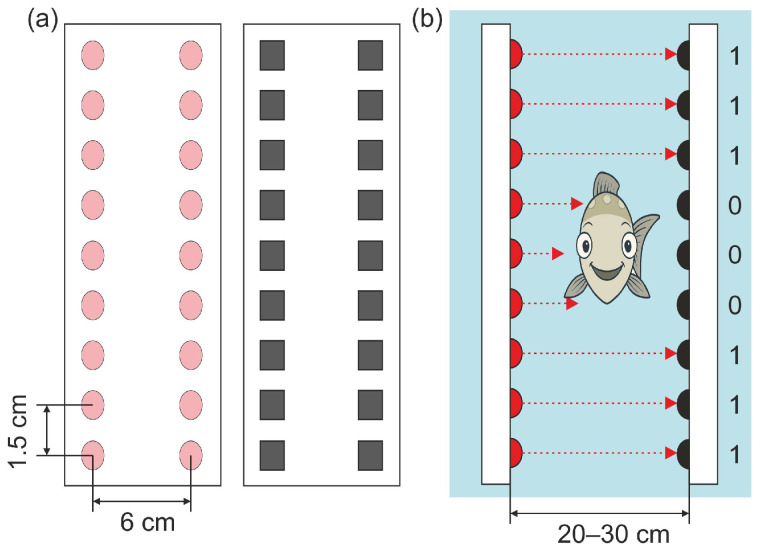
Working principle of an infrared (IR) beam-break fish counter. (**a**) Vertical array of IR emitters (left) and receivers (right) creating an invisible light curtain across the passage channel. (**b**) As a fish crosses the curtain, it interrupts specific IR beams, generating a binary pattern of beam-break events.

IR counters operate on the principle of detecting objects that interrupt a structured field of IR light. Typically, the system comprises two arrays of IR emitters (LEDs) aligned with corresponding receivers (IR photodetectors) to form a “light curtain”. When a fish passes through the detection zone, it breaks one or more IR beams (Figure 1). These interruptions are registered as binary signals (outputting a digital ’0’ for a broken beam and ‘1’ for an intact beam), which are then processed to reconstruct the fish’s silhouette and infer its movement characteristics. The IR technology is especially suited to fish passage monitoring because infrared light is invisible to fish and non-intrusive to behavior [12].

**Figure 2:**
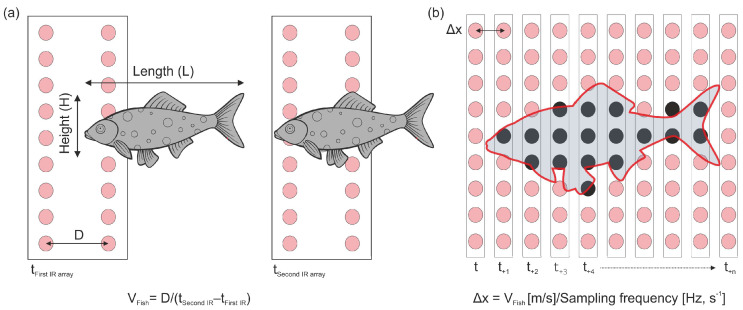
Passage process and silhouette reconstruction in an infrared (IR) beam-break fish counter. (**a**) Fish enters the first IR array at time t_First IR array_ and enters the second array at time t_Second IR array_, allowing estimation of swimming speed. (**b**) Sequential beam interruptions of an array across time intervals are compiled into a silhouette. The distance between IR samples of the same array (Δx) is based on the sampling frequency and swimming speed.

This temporal sequence of beam interruptions across the two parallel arrays allows for the extraction of key passage metrics:

- **Direction of Travel:** Determined by the order in which the first and second arrays are sequentially interrupted.
- **Passage Speed (V_fish_):** In Fish Tracker, this is calculated from the time delay (t_second IR array_ − t_first IR array_) between the initial interruption of corresponding points on the first and second arrays and the known distance (D) between the arrays (V_fish_ = D/(t _second IR array_ − t_first IR array_)) (Figure 2a). A similar approach is applied at the exit of the silhouette, yielding two velocity estimates per event.
- **Fish Silhouette and Size Estimation:** Once passage speed is determined, the sequence of sensor states (broken/intact beams), recorded at a constant sampling frequency (e.g., 50 Hz), allows for spatial reconstruction to estimate fish size (Figure 2b). Unlike traditional methods that estimate length from the measured maximum height using a known species-specific allometric relationship (length–height ratio), which often introduces bias when the species is unknown, the FishTracker system uses a direct reconstruction approach. It maps the vertical profile of broken beams (fish height at each time step) against the calculated horizontal distance travelled between beam samples (Δx = V_fish_/Sampling Frequency) to generate a 2D silhouette. Length and height are then estimated from this reconstructed silhouette, reducing dependency on species-specific ratios.

Before any silhouette reconstruction or parameter estimation, it is essential to first detect valid passage events from the stream of binary signals (0’s and 1’s). This is achieved by applying a sequence of logical filters during data processing to distinguish true fish passages from false positives caused by debris, noise, or incomplete entries. A valid event typically requires a continuous and directional sequence of beam interruptions—beginning with the upstream sensor array and concluding only after the downstream array is fully cleared. The detailed implementation of this filtering logic is described in Section 2.4 (Software architecture).

### 2.3. Hardware

The Fish Tracker system is based on a modular infrared beam-break design, composed of two main units: the IR curtain (sensor unit) and the data management unit. The sensor unit is rated IP68 for underwater operation, while the processing and communication module is housed in an IP65-rated enclosure.

**Figure 3:**
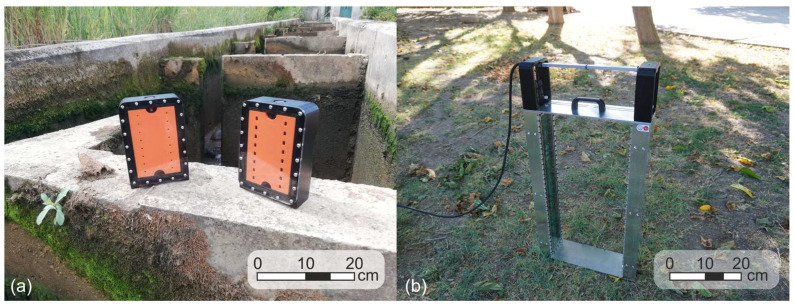
Two Fish Tracker prototypes. (**a**) The Fish Tracker Mini, an initial prototype with 8 IR photodetectors per array, which was used for early functional testing and alignment verification. (**b**) The final Fish Tracker system (full version), featuring 40 IR photodetectors per array and full-scale structural dimensions, enclosed in waterproof aluminum housing for field deployment.

During the design phase, two main prototypes were developed: the Fish Tracker Mini, which featured 8 IR photodetectors per array and served as a platform for configuration testing, and the final Fish Tracker prototype, which includes 40 IR photodetectors per array and reflects the full-scale system dimensions (Figure 3).

#### 2.3.1. Sensor Unit: IR Curtain

The core detection module consists of two opposing vertical arrays: an emitter array of infrared (IR) LEDs (OSRAM SFH 4550) and a receiver array of IR photodetectors (Vishay SFH 203). Each array contains 8 elements in the mini version and 40 elements in the full-scale version, arranged in two vertical lines with 1.5 cm spacing between adjacent elements. This configuration provides a total detection height of 60 cm in the full-scale version. The emitter and receiver arrays are positioned 6 cm apart. This inter-array spacing is critical, as it defines the minimum fish length required for the Fish Tracker system to reliably register a complete passage and estimate parameters. Furthermore, this 6 cm distance, in conjunction with the spacing of elements within each array, directly influences the overall system resolution and the likelihood of false positives or overlapping detections.

Each IR emitter (850 nm) signal is detected by the corresponding IR receivers. When a fish passes through the sensing zone, it interrupts one or more beams. The receiver array registers these interruptions as binary signals—‘0’ for a broken beam, ‘1’ for an intact beam—and transmits the full matrix of beam states at a fixed sampling rate of 50 Hz via serial communication to the central processing unit.

In the full-scale system, 80 digital inputs (40 beams × 2 lines) are managed using five I/O expanders (MCP23017, Microchip Technology Inc., Chandler, AZ, USA) connected via SPI to a single ESP32-WROOM-32 microcontroller programmed in the Arduino IDE (version 2.3.2). Each MCP device is assigned a unique address, allowing synchronized acquisition and communication of the entire sensor state. Custom printed circuit boards (PCBs) for both emitter and receiver arrays were designed using KiCad open-source software (version 5.0.1).

The emitter and receiver units are enclosed in CNC-machined housings: the mini prototype used POM (polyoxymethylene), while the final prototype uses aluminum for greater mechanical stability. Although POM was initially considered for the full-scale version due to its machinability and weight advantages, deformation issues (bending) during assembly led to the decision to switch to aluminum. POM remains a preferred material and may be reconsidered in future versions with optimized design and wall thickness. Transparent polycarbonate covers sealed with O-rings ensure watertight operation in both configurations. All CNC-machined parts were designed using Autodesk Fusion 360 (version 2602.1.25). Additionally, the system was designed for easy deployment and adaptability. For instance, a series of threaded holes at various heights and positions provides flexibility for mounting in different configurations.

**Figure 4:**
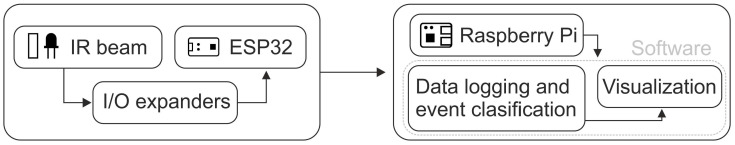
Overview of the Fish Tracker system architecture. Infrared beam interruptions are processed by an ESP32 microcontroller with I/O expanders, which transmits the data to a Raspberry Pi. The Raspberry Pi performs data logging, event classification, and visualization within a modular software environment.

The receiver unit generates a continuous stream of digital data representing beam states, which is transmitted in real-time to the data management unit for processing and storage (Figure 4).

#### 2.3.2. Data Management Unit

The data management unit is built around a Raspberry Pi 5 single-board computer (Model B Rev 1.0 of 8 GB RAM), which receives and processes the binary data stream from the ESP32 controller and runs on Raspbian OS (64-bit, Bookworm). It performs key functions including local processing, data storage, and system communication.

Power is supplied by a regulated DC power source capable of delivering up to 13.1 V and 10 A. This source feeds both the Raspberry Pi—via a 5 V regulator—and the emitter and receiver arrays through a DC buck converter up to 12 V. While the receiver unit includes its own onboard 5 V voltage regulator, the emitter receives its voltage directly from the buck converter output.

The system includes a touchscreen interface connected to the Raspberry Pi for local interaction and visualization of the graphical user interface (GUI). This interface facilitates field diagnostics and system configuration. A cellular SIM modem is also integrated, enabling remote access, data upload, and system monitoring via mobile networks.

All external connectors and power lines are IP68-rated or higher, ensuring durability and waterproofing in harsh environmental conditions typical of field deployments.

The total cost of all components for the Fish Tracker system was kept under 2000 EUR. This includes approximately EUR 1400 for the IR curtain—comprising emitters, receivers, CNC-machined housings, and control electronics—and around EUR 600 for the data management unit, which includes the Raspberry Pi, touchscreen interface, SIM modem, power regulation system, cabling, and waterproof enclosure.

### 2.4. Software Architecture and Data-Processing Logic

The Fish Tracker software (version 1.0) system is designed as a modular set of Python scripts deployed on a Raspberry Pi. Its primary functions include sensor data acquisition, fish passage detection, event filtering, silhouette reconstruction, and user interaction via a graphical interface.

#### 2.4.1. Script Structure and Interaction

**Figure 5:**
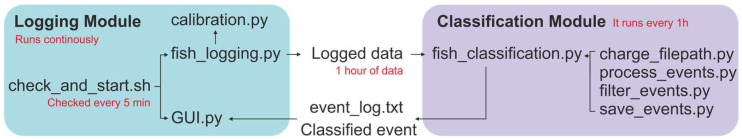
Diagram of the Fish Tracker software architecture. The logging module runs continuously, saving hourly binary data and performing real-time calibration. The classification module, executed every hour, processes the logged data to extract and validate fish passage events using a series of interconnected scripts.

The software is organized into two main operational modules: a continuous Logging Module and an hourly Classification Module (Figure 5). The Logging Module is responsible for acquiring binary data from the IR sensor arrays through an ESP32 microcontroller (fish_logging.py). This data, which records the status of each beam (0 for interrupted and 1 for uninterrupted), is stored in hourly text files. Before recording begins, a calibration script (calibration.py) is triggered to ensure that all the beams are correctly transmitting and receiving data. To guarantee continuous operation, a watchdog script (check_and_start.sh) is executed every five minutes via a cron job, automatically restarting the logger and the GUI if either process stops unexpectedly.

Once a new hour of data has been logged, the Classification Module (fish_classification.py) begins its routine. It accesses the most recent log file and identifies candidate fish passage events based on sequences of beam interruptions. These events are processed using a chain of scripts that load the raw data, apply filtering criteria, and extract key parameters. Validated events are saved as structured files (individual clips corresponding to each event) in a classified event folder. A central log file (event_log.txt) stores summary information for each event, including passage direction, velocity, estimated length, and height.

#### 2.4.2. Event Detection and Filtering Logic

Event detection in Fish Tracker begins by segmenting the binary stream of sensor data to identify discrete passage events. This is achieved using the process_fish_events () function, which analyzes the data for structured interruptions—typically characterized by accumulations of ‘0’s (interrupted beams) separated by ‘1′s (intact beams)—that indicate potential fish movement.

To enhance detection reliability, a prototype filtering step, implemented by the remove_random_lines () function, eliminates spurious signals. These signals often manifest as short, isolated beam interruptions—characterized by minimal vertical extent (i.e., ‘lines of reduced width’) and a lack of corresponding detection patterns across both sensor arrays—which are typically attributed to environmental noise. Once cleaned, the direction of each event is estimated by comparing the order and timing of beam activations across the upstream and downstream sensor arrays (process_fish_directions ()).

Subsequently, the system calculates the swimming velocity of each potential fish using the known 6 cm distance between arrays and the time difference between entry and exit across the beam curtain (process_fish_velocities ()). This velocity is then used in conjunction with the event duration to estimate fish length (process_fish_length ()), while body height is inferred from the vertical extent of beams interrupted during the passage (process_fish_height ()). These processing functions yield two distinct values for their respective parameters.

To refine detections, a key filtering step is executed using the filter_events () function. This function evaluates each candidate event using three criteria: directionality consistency, velocity plausibility, and morphological coherence. First, it checks that the sum of the inferred directions at the start and end of an event is non-zero, indicating a clear passage rather than oscillating or indecisive motion. Second, it filters out events with a mean swimming velocity of zero, as such cases typically indicate stationary debris or invalid readings. Third, it eliminates events where the estimated body height is below a biologically plausible threshold (e.g., 1.5 cm), a common sign of environmental noise or partial detections. These combined criteria help retain only those events that most likely represent real fish passages while discarding ambiguous or noisy data.

Finally, a timestamp is assigned to each valid event (date_time_calculation ()), and a summary of its parameters (date, direction, velocity, length, height) is saved in a central log file (event_log.txt). Simultaneously, the raw binary data for each individual event is saved in a structured folder (classifiedEvents) for future processing or review.

**Figure 6:**
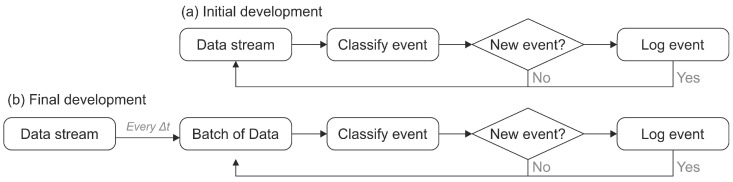
Comparison of classification algorithms used in the Fish Tracker system. (**a**) Original implementation: real-time classification and logging were performed on each incoming data point, demanding high processing loads. (**b**) Optimized implementation: data are collected in time-based batches and processed asynchronously, improving reliability and allowing for error filtering before logging.

Initially, the detection algorithm was developed to operate in real time on a more powerful PC (Figure 6a). It relied on a logical decision tree to distinguish true fish passages from confounding elements such as debris or fish turning around mid-passage. However, this approach proved inefficient when ported to the lower-processing-capacity Raspberry Pi. As a result, the methodology was redesigned into a stepped classification workflow that leverages pre-processed indicators to identify valid fish passages (Figure 6b). This revised structure significantly improved performance, reducing computational load and enabling robust, scalable field deployment.

The classification algorithm, which includes a graphical implementation for visualizing results, along with the experimental data from this study, is available at ZENODO (https://doi.org/10.5281/zenodo.15501832).

#### 2.4.3. GUI and Visualization

**Figure 7:**
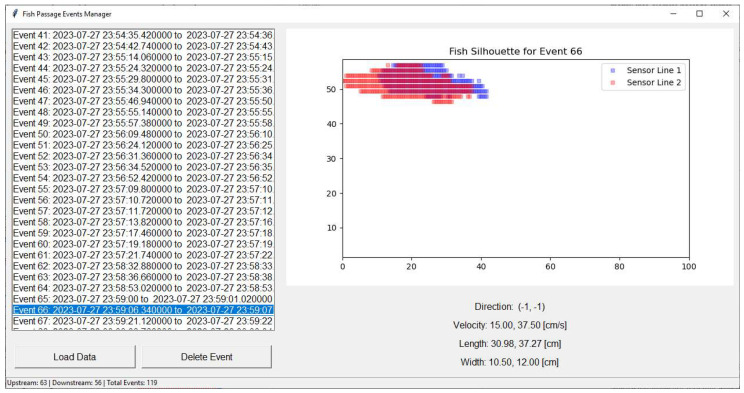
Screenshot of the Fish Tracker graphical user interface (GUI), developed using Tkinter and Matplotlib. The GUI allows real-time interaction for browsing detected events, inspecting reconstructed fish silhouettes, reviewing associated metadata (direction, velocity, length, and height), and manually managing recorded data.

Once the events have been classified, the system includes a user-friendly GUI built with Tkinter (version 8.6.14) and integrated with Matplotlib (version 3.9.2) for plotting. This interface allows users to browse detected events, view reconstructed fish silhouettes, inspect event metadata (e.g., direction, velocity, estimated dimensions), and manually delete erroneous records (Figure 7). Data can be refreshed in real time, offering an efficient and interactive tool tailored for both field monitoring and research applications.

### 2.5. Experiments

To validate the Fish Tracker system, three sets of experiments were conducted. The first experiment served as a proof of concept using dummy fish replicas made of cardboard. These experiments aimed to test the basic functioning of the system, including the ability to detect passage direction and estimate fish size. Two dummy sizes (13 cm and 16.5 cm in length and both 6.4 cm in height) were passed through the system in both directions (10 repetitions per direction, 20 in total). This allowed evaluation of detection rates, average length estimates, and standard deviations, providing a benchmark for system performance at controlled dimensions.

**Figure 8:**
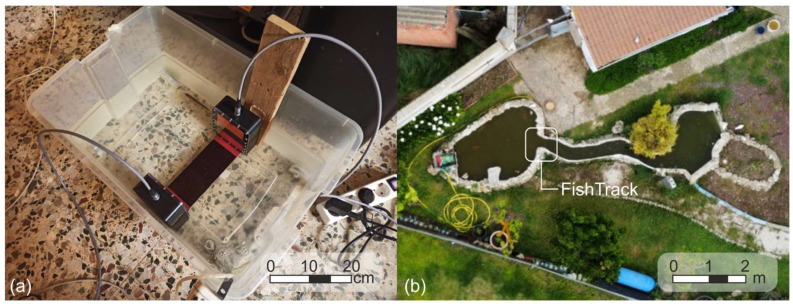
Layout of the experiments. (**a**) Set up for live fish tests with bleak under controlled turbidity. (**b**) Experimental configuration in the koi carp pond.

The second experiment involved n = 2 live fish, the bleak (*Alburnus alburnus*), an invasive alien species common in Iberian rivers. These specimens were selected due to their small body size, which lies near the lower detection limit of the system (approximately 10 cm in length and 1.5 cm in height). The tests were conducted in a calm-water pond under two turbidity conditions: low turbidity (1.77 NTU) and moderate-high turbidity (14.75 NTU) (Figure 8a). Turbidity was measured with an AquaFlour^®^ Turbimeter (Turner Designs Inc., San Jose, CA, USA). In each configuration, at least 10 passage events per fish were recorded and manually validated. Average length, standard deviation, and detection rates were computed to assess system performance under realistic and challenging field conditions.

Both tests were run using a pre-release version of the software, with observers present to annotate and compare real-time detections against visual confirmation.

Fish handling followed EU (Directive 2010/63/UE) and Spanish regulations (RD53/2013 and RD118/2021) for animal welfare under experimentation, with a specific project (7904309) approved by the competent authority (Junta de Castilla y León).

A third complementary experiment was conducted in a shallow pond with 20 koi carp (*Cyprinus carpio*) (Figure 8b). The system lasted 1.5 h without disturbing the fish, simulating a natural deployment scenario. The water depth was around 15 cm. A Sony Alpha ZV-E10L camera (Sony Group Corporation, Tokyo, Japan) was set up to continuously record the experiment. Raw data and video footage were later analyzed in the lab to assess fish behavior and passage alignment.

### 2.6. Data Management and Analysis

For the first two experiments (dummy fish and bleak trials), event data were manually annotated using an early version of the graphical user interface (GUI). The estimated length, body height, and number of detected events were extracted from each passage. These values were used to compute the mean, standard deviation, and detection success rate, defined as the ratio of detected events to total simulated or observed passages.

In the third experiment, performed under semi-natural conditions with koi carp, an external validation process was implemented using independent video recordings. A high-definition camera continuously recorded the fish passage zone, and an operator manually reviewed the footage to identify and timestamp each passage. These manual annotations served as the ground truth for comparison with the detections recorded by the Fish Tracker system.

Each system-detected event was categorized as either a *hit* (true positive) or a *miss* (false negative) based on whether it corresponded to a verified fish passage. In the case of mismatches, events were further analyzed using the IR sensor activation matrix in combination with video data. Errors were classified into the following categories:

- (I) Single fish not detected due to distortion in the water surface,
- (II) Second fish passage not detected due to surface distortion caused by a preceding fish,
- (III) Multiple fish overlapping within the detection curtain,
- (IV) Self-cancellation due to bidirectional movement within the sensing zone.

Representative video clips of each error type are included as Supplementary Materials.

The system estimates both body height and length, which are essential metrics for population-level analyses. While height can be directly inferred from the number of vertically interrupted infrared beams, length estimation relies on novel indirect approaches (Figure 2). Therefore, although no fish were physically measured during the final trial, a preliminary assessment was conducted to evaluate whether the height estimation method could yield biologically plausible estimates under real-world conditions. For this, we compared Fish Tracker outputs with published allometric models. In particular, we employed the standard allometric formula *L* = *b* ⋅ *H* where *L* is fish length, *H* is body height, and *b* is a proportionality constant. Literature data from cultured common carp populations—known for their lower *L*:*H* ratios (typically between 2.5 and 3.0 for cultivated strains)—were used as the reference model [25].

For all events considered accurate (i.e., not affected by overlap or water surface distortion), the lengths calculated from height data using the allometric formula were compared with those directly estimated by the Fish Tracker system.

All the analyses and programming were performed using Python 3.10 with the Spyder IDE (Version: 5.5.1), employing standard scientific libraries such as NumPy, pandas, and Matplotlib. This comparison allowed an objective assessment of agreement between both estimation methods, facilitating future calibration and model refinement.

## 3. Results

### 3.1. Preliminary Test

The first phase of validation focused on assessing the performance of the Fish Tracker system under controlled conditions using both dummy fish models and live specimens (bleak). Two dummy fish sizes (13.0 cm and 16.5 cm in length) were passed through the detection system to evaluate its accuracy in both counting and estimating fish length.

**Figure 9:**
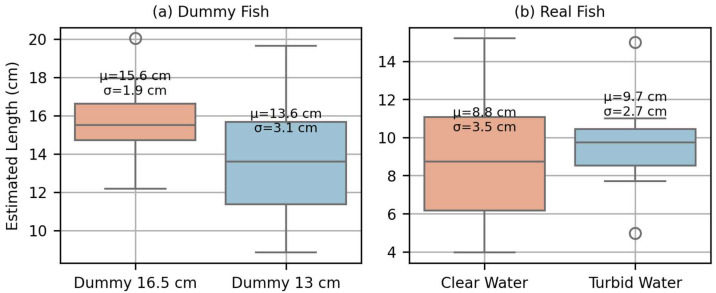
Estimated fish lengths recorded by the Fish Tracker system during validation trials (μ = mean and σ = standard deviation). (**a**) Boxplot showing estimated lengths for dummy fish of known sizes (13.00 cm and 16.50 cm). (**b**) Boxplot showing estimated lengths for live fish (bleak) under two turbidity conditions: clear water (1.77 NTU) and turbid water (14.75 NTU).

The detection rate was 100% for the 16.5 cm dummy fish and 95% for the 13.0 cm dummy fish, indicating a slight drop in sensitivity near the system’s lower detection threshold. Figure 9a illustrates the distribution of estimated lengths. The system yielded mean length values close to the actual sizes, although variability increased for the smaller individuals—highlighting the influence of fish size on measurement consistency.

In terms of height estimation, the system recorded average values of 5.59 ± 1.37 cm for the 13.00 cm dummy and 5.74 ± 1.71 cm for the 16.50 cm dummy. These results are consistent with the actual measured heights of dummy fish (6.4 cm), confirming the system’s capacity to capture relevant morphometric dimensions with acceptable accuracy.

In the live fish trials with bleak (Figure 9b), events were recorded under two turbidity conditions: low (1.77 NTU) and medium (14.75 NTU). Despite this modification, no significant differences were observed in the number of detected events or the distribution of estimated lengths between the two conditions. The overall detection rate was slightly reduced (85%) when compared to dummy tests, reflecting a decrease in performance with smaller fish sizes.

The system recorded average height values of 0.60 ± 0.65 cm under low turbidity and 0.58 ± 0.92 cm under medium-high turbidity. Based on these results, and to account for potential underestimation due to partial beam occlusions, the detection algorithm was subsequently modified. Specifically, an additional half of the vertical beam spacing was added to the estimated height, as previously, only one activated beam bin was interpreted as indicating zero height. This adjustment was introduced to improve height estimation for small-bodied fish near the system’s detection threshold (10 cm in length, ~1.5 cm in height).

### 3.2. Final Test

#### 3.2.1. Counting Rates

The performance of the Fish Tracker system was further evaluated under semi-natural conditions by comparing its automatic detections—based on the final software architecture—with manual annotations obtained from synchronized video recordings.

The overall error rate in fish detection was 30.97%, a value largely influenced by the experimental setup and the distortions of the water surface during the tests. A detailed review of all mismatches between system detections and manual observations allowed the classification of error sources into four main categories (Table 1). This analysis provides critical insights into the system’s current limitations and potential areas for improvement.

As shown in Table 1, the most frequent source of error (45.71%) was related to distortions in the water surface caused by the passage of fish. These disturbances interfered with beam transmission and detection, resulting in incomplete or misinterpreted events. In 31.43% of cases, a second fish was not detected, masked by surface distortion generated by a preceding individual.

Overlapping fish accounted for 20.00% of the errors, emphasizing the difficulty of resolving closely spaced individuals passing simultaneously. Lastly, 2.86% of the errors were attributed to self-cancelling events—situations where bidirectional movement or erratic fish behavior near the detection area appeared to negate valid detection sequences.

**Figure 10:**
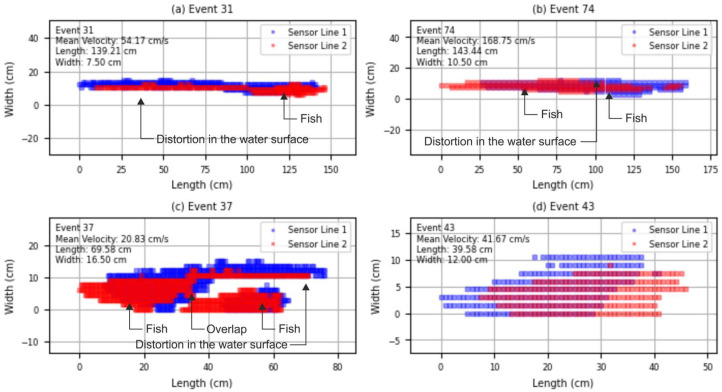
Examples of reconstructed fish silhouettes from the Fish Tracker system, showing different event outcomes. (**a**) Single fish not detected due to distortion in the water surface (case I). (**b**) Second fish passage not detected due to surface distortion caused by a preceding fish (case II). (**c**) Multiple fish overlapping within the detection curtain (case III). (**d**) Normal fish passage with consistent detection from both sensor lines (case V). Videos of these events are provided as Supplementary Materials, including an example of Case IV.

To better illustrate the nature of the classification errors described above, representative examples of reconstructed fish passage events are shown in Figure 10. These visualizations, derived from the raw beam-interruption matrices, depict different error scenarios frequently encountered during the validation trials. Each graph corresponds to a distinct case of misdetection or abnormal passage, providing insight into how specific conditions—such as water distortion, fish overlap, or atypical movement—affect the silhouette reconstruction process and ultimately the accuracy of the system. Additionally, a video recording of the representative events shown in Figure 10 is provided as Supplementary Materials.

#### 3.2.2. Morphometrics

One of the primary objectives of the Fish Tracker system is to characterize fish and their movement over time without the need for physical handling or tagging.

**Figure 11:**
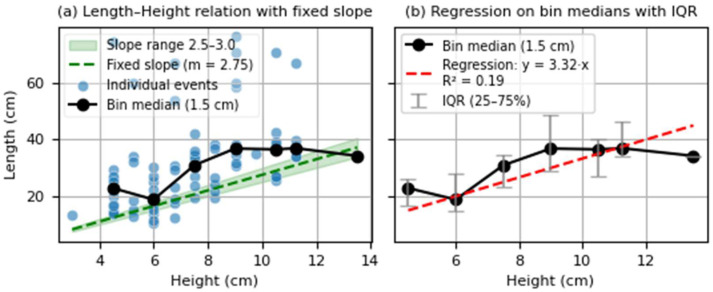
Scatterplots of estimated fish lengths and heights. (**a**) Individual valid detection events are shown, along with reference lines corresponding to fixed allometric length-to-height ratios (*L* = 2.5*H* and *L* = 3.0*H*), and medians per 1.5 cm height bin. (**b**) Linear regression fitted to the bin medians, with interquartile range (IQR) error bars. Only detection events not affected by overlapping, water surface distortion, or laminar artifacts were included in the analysis.

Morphometric validation analysis demonstrates that, for fish events unaffected by overlap or water surface distortion, the Fish Tracker counter exhibits strong internal consistency in its dimensional measurements. A comparison between direct length measurements and those derived from height using an allometric model shows a high degree of agreement, indicating that the sensors and system processing consistently capture related dimensional information under the experimental setup (Figure 11).

As shown in the figure, although several observations deviate from the expected allometric range, the highest concentration of data points aligns closely with the literature-based linear relationships.

## 4. Discussion

### 4.1. Hardware and Software

The software architecture of the Fish Tracker system performed reliably during field trials. However, additional deployments are needed to thoroughly evaluate long-term performance, robustness, and comparison with other fish-counting technologies. Such evaluations are planned in fishways within the Kantauribai LIFE Project (LIFE21-NAT-ES-LIFE KANTAURIBAI (101074197)). The current software employs an hourly classification approach specifically designed to minimize computational demands, facilitating its operation on low-power, single-board computers such as the Raspberry Pi. While earlier software versions operated in real time, these proved overly demanding for limited computational resources.

At the core of the system is the fish classification algorithm, which has demonstrated reliable performance except in cases generating signals outside logical expectations, where purely logic-based approaches inevitably fail. Future improvements could incorporate convolutional neural networks (CNNs) to automatically detect complex events, anomalies, or previously identified errors, contingent upon the availability of robust validation datasets. Currently, the classification algorithms and datasets from this study are publicly available on Zenodo (https://doi.org/10.5281/zenodo.15501832), although the existing dataset size remains limited for training advanced machine learning models.

To improve the robustness of the classification algorithm—particularly for downstream movement or debris-related events—it may be necessary to include additional validation tests using common drifting materials such as leaves, twigs, or plastic bags. Such objects can occasionally mimic fish-like interruptions in beam data, especially under turbulent conditions, and should be considered in future calibration experiments.

A key innovation of the Fish Tracker system lies in its length estimation approach. Rather than applying predefined species-specific allometric relationships (e.g., length–height ratios), Fish Tracker estimates length directly from passage velocity and silhouette duration using the beam array data. This species-independent methodology provides biologically plausible estimates across different species and sizes, making it well suited for multispecies environments or locations where biometric references are unavailable [13,26]. To the best of our knowledge, this represents a novel contribution in the domain of fish passage monitoring. Nevertheless, species-specific allometric relationships can also be easily incorporated into the system if required.

An important advantage of Fish Tracker is its reliance on open-source technologies, significantly reducing the material cost to below EUR 2000 (production and installation costs < EUR 10.000). This affordability enables broader adoption, particularly in contexts where conventional high-cost technologies are prohibitive. Following the original ethos behind low-cost, accessible technologies like the Raspberry Pi, testing protocols are openly shared to promote wider implementation and adaptation.

Moreover, the modular design and compact dimensions of Fish Tracker facilitate easy transport and rapid installation in diverse field settings. Its open software architecture enables straightforward integration with other open-source sensor platforms based on similar technologies (e.g., Arduino, Raspberry Pi), promoting interoperability and system scalability for broader environmental monitoring applications [27].

### 4.2. Performance and Limitations

Detection accuracy clearly depended on fish size, with smaller fish (10.00 cm and 13.00 cm lengths) detected less reliably compared to larger specimens (16.50 cm). This limitation primarily arises from beam spacing constraints (in the current configuration, the smallest detectable fish is 3 cm in height and 6 cm in length), similar to limitations observed in commercial systems such as RiverWatcher, which specifies a minimum recommended detection size of around 4 cm fish height [28]. Increasing LED density or reducing beam spacing could theoretically enhance small-fish detection; however, this poses risks of overlapping beams and distorted shape reconstructions. Laser-based systems were considered during preliminary tests but ultimately discarded due to alignment complexities, reduced durability, and higher emitter costs.

In semi-natural scenarios, Fish Tracker correctly classified approximately 70% of passages, comparable to commercial systems like RiverWatcher, which report detection accuracies ranging from 56% to 84% [29], 76% [30], 64% for fish between 30 and 40 cm [31], and 32% for fish between 34 and 52 cm [28]. However, in our trials, most errors (45.71% + 31.43%) arose from partial submersion, causing water surface distortions. Fully submerging the system and ensuring proper hydrodynamic installation could significantly mitigate these issues. During field installations in fishways, additional potential sources of error—such as turbulence-induced bubbles, as noted in previous studies [12]—should be carefully considered and mitigated during system setup.

Other error types, such as multiple fish passages and overlapping fish, commonly reported in commercial systems [13,30], were also observed with Fish Tracker, highlighting inherent technological limitations shared across infrared-based counting systems. Additionally, other potential sources of error not explored in this study may include the passage of non-target animals (e.g., otters, mink, cormorants, or water voles) or floating debris, which could trigger false detections or distort signal interpretation. Future deployments should consider these possibilities in both system design and error classification protocols.

Length estimation demonstrated reasonable consistency despite inherent constraints. Unlike commercial systems relying on species-specific biometric ratios, Fish Tracker estimates length based on passage velocity and beam-interruption duration, a novel species-independent method. Although significant variation was noted (Figure 11), mean length estimates proved sufficiently accurate for population-level assessments. Comparative studies indicate that commercial systems such as RiverWatcher often underestimate fish lengths [29,32].

It is important to note that factors such as fish swimming angle and local water velocity may influence detection quality. The Fish Tracker system assumes that fish move horizontally and perpendicularly through the IR curtain. However, angled or erratic passages—particularly under turbulent flow—could distort the silhouette reconstruction and affect length and velocity estimates. While not quantified in this study, these effects are acknowledged as potential sources of error and will be examined in future field trials.

In still or low-flow waters, fish may vary their swimming speed more freely, potentially introducing noise into velocity and length estimates. However, given the short detection zone and typical installation in narrow passages, such effects are expected to be minor. It remains possible that some individuals cross the array at an angle, leading to a slight underestimation of body height. This is currently under evaluation in ongoing experiments.

Fish Tracker offers considerable advantages as a non-contact system, including reduced operational costs and minimal fish disturbance, critical for adaptive management practices. Its modular, open-source base design significantly lowers the material costs, making widespread deployment feasible. However, successful integration requires specialized hydraulic engineering expertise to maintain effective water flow dynamics inside a fishway.

Compared to camera-based systems, Fish Tracker requires significantly lower maintenance and power consumption, making it suitable for remote, solar-powered setups. While regular cleaning is necessary, we recommend performing it at least once per month. Maintenance schedules can be efficiently managed by monitoring IR emitter performance. Challenges in IR counters seem to remain in accurately detecting overlapping fish and achieving species-level identification. Camera systems offer the distinct advantage of species identification if well-trained and provide more visually compelling outputs, which can be a persuasive factor for managers. In this regard, given that the error sources differ between these two types of systems, their combination could be a promising approach to achieve even more robust and comprehensive monitoring results. A hybrid system, already implemented in some commercial systems, could also reduce video processing time by using IR-based detections to guide or trigger targeted video analysis.

## 5. Conclusions

This study successfully details the design, development, and initial validation of Fish Tracker, a novel low-cost, open-source-based infrared beam-break counter aimed at enhancing the accessibility of fish passage monitoring. Initial validation confirmed its operational viability, demonstrating high detection accuracy (95–100%) under controlled conditions. In semi-natural settings, a detection rate of approximately 70% was achieved, with this performance notably influenced by factors such as water surface distortions arising from the partial submersion of the unit during experimental setups.

Fish Tracker’s core contributions lie in its partially open-source-based architecture and significantly reduced material cost (EUR < 2000—production and installation costs < EUR 10.000), which, combined with its non-contact nature and minimal fish disturbance, make widespread deployment for adaptive management practices more feasible [27]. Its modular design facilitates lower operational demands compared to many commercial systems, particularly suiting remote, solar-powered setups. Furthermore, the innovative species-independent approach to deriving fish morphometrics from passage speed and beam-interruption data presents a versatile tool for multispecies contexts.

While Fish Tracker offers these advantages, certain limitations are acknowledged. Successful hydraulic integration is crucial for optimal performance, and like other infrared-based systems, challenges persist with very small fish, species-level identification, and the accurate detection of overlapping individuals. Regular cleaning, though manageable via IR emitter performance monitoring, also remains a practical consideration.

Future research should focus on extended field studies across diverse environmental conditions and direct comparisons with established commercial systems to further refine performance and validate long-term operational reliability. Algorithm enhancements, potentially incorporating machine learning techniques, are also anticipated to improve accuracy in complex scenarios and expand applicability. In parallel, additional laboratory experiments using smaller-bodied fish replicas (approximately 3 cm) are planned to refine the system’s detection threshold and assess variability at the lower size limit. Further tests will also explore the effect of increasing or reducing the density of infrared diodes on detection accuracy and spatial resolution. Additionally, integrating an optional hybrid configuration with a camera module could provide species-level identification capabilities and enhance acceptance among managers and stakeholders.

Ultimately, Fish Tracker represents a significant step towards more accessible and cost-effective fish passage monitoring. It offers a promising tool to support broader and more effective evaluation of fishway performance, thereby empowering better-informed river management and conservation efforts, especially in locations where economic constraints currently limit the adoption of advanced monitoring technologies.

## Figures and Tables

**Table 1:** Classification of counting errors observed during the final validation experiment.

Type of Error	Percentage (%)
One fish—distortion in the water surface (case i)	45.71
Second fish—distortion in water sheet (case ii)	31.43
Multiple fish overlapping (case iii)	20.00
Self-cancelling (case iv)	2.86

## Data Availability

The datasets, classification scripts, and Supplementary Materials generated and used in this study are openly available in Zenodo at https://doi.org/10.5281/zenodo.15501832.

## Supplementary Materials

The following supporting information can be downloaded at: https://www.mdpi.com/article/10.3390/s25134112/s1, Video S1: Case I, Video S2: Case II, Video S3: Case III, Video S4: Case IV, Video S5: Case V.

## Abbreviations

The following abbreviations are used in this manuscript:

IR	Infrared
PCBs	Printed Circuit Boards
POM	Polyoxymethylene
GUI	Graphical User Interface
IQR	Interquartile Range
CNNs	Convolutional Neural Networks

## References

[1] Lucas M., Baras E. (2001). Migration of Freshwater Fishes.

[2] Nilsson C., Reidy C.A., Dynesius M., Revenga C. (2005). Fragmentation and Flow Regulation of the World’s Large River Systems. Science.

[3] Yu Y., Chang J. (2025). Preliminary Analysis of the Construction and Operation Status of Fish Passage Facility in China. Ecol. Eng..

[4] Hatry C., Binder T.R., Thiem J.D., Hasler C.T., Smokorowski K.E., Clarke K.D., Katopodis C., Cooke S.J. (2013). The Status of Fishways in Canada: Trends Identified Using the National CanFishPass Database. Rev. Fish Biol. Fish..

[5] Bunt C.M., Castro-Santos T., Haro A. (2016). Reinforcement and Validation of the Analyses and Conclusions Related to Fishway Evaluation Data from Bunt et al.: “Performance of Fish Passage Structures at Upstream Barriers to Migration.”. River Res. Appl..

[6] European Commission (2000). Directive 2000/60/EC of the European Parliament and of the Council Establishing a Framework for the Community Action in the Field of Water Policy.

[7] Travade F., Larinier M. (2002). Monitoring Techniques for Fishways. Bull. Fr. Pêche Piscic..

[8] Jepsen N., Thorstad E.B., Havn T., Lucas M.C. (2015). The Use of External Electronic Tags on Fish: An Evaluation of Tag Retention and Tagging Effects. Anim. Biotelem..

[9] Thiem J.D., Taylor M.K., McConnachie S.H., Binder T.R., Cooke S.J. (2011). Trends in the Reporting of Tagging Procedures for Fish Telemetry Studies That Have Used Surgical Implantation of Transmitters: A Call for More Complete Reporting. Rev. Fish Biol. Fish..

[10] Clemens B.J., Matley J.K., Klinard N.V., Lennox R.J., Sortland L.K., Cooke S.J. (2023). The Need for Reporting Rationale and Detailed Methods in Studies That Surgically Implant Fish with Electronic Tracking Devices. Fisheries.

[11] Bravo-Córdoba F.J., García-Vega A., Fuentes-Pérez J.F., Fernandes-Celestino L., Makrakis S., Sanz-Ronda F.J. (2023). Bidirectional Connectivity in Fishways: A Mitigation for Impacts on Fish Migration of Small Hydropower Facilities. Aquat. Conserv. Mar. Freshw. Ecosyst..

[12] Fewing G.A., Trust A.S. (1993). Automatic Salmon Counting Technologies—A Contemporary Review.

[13] Haas C., Thumser P.K., Hellmair M., Pilger T.J., Schletterer M. (2024). Monitoring of Fish Migration in Fishways and Rivers—The Infrared Fish Counter “Riverwatcher” as a Suitable Tool for Long-Term Monitoring. Water.

[14] European Commission (2020). EU Biodiversity Strategy for 2030: Bringing Nature Back into Our Lives.

[15] Fuentes-Pérez J.F., Bravo-Córdoba F.J., García-Vega A., Eckert M., Branco P., Sanz-Ronda F.J. (2024). The Effect of Hydrological Variability on Stepped Fishways. J. Hydrol..

[16] Nowak L.J., Lankheet M. (2023). Understanding and Optimizing Fish Counting Techniques Based on Electrical Impedance Measurements. PLoS ONE.

[17] Maxwell S.L., Gove N.E. (2007). Assessing a Dual-Frequency Identification Sonars’ Fish-Counting Accuracy, Precision, and Turbid River Range Capability. J. Acoust. Soc. Am..

[18] Martignac F., Baglinière J.-L., Ombredane D., Guillard J. (2021). Efficiency of Automatic Analyses of Fish Passages Detected by an Acoustic Camera Using Sonar5-Pro. Aquat. Living Resour..

[19] Wei Y., Duan Y., An D. (2022). Monitoring Fish Using Imaging Sonar: Capacity, Challenges and Future Perspective. Fish Fish..

[20] Dunkley D.A., Shearer W.M. (1982). An Assessment of the Performance of a Resistivity Fish Counter. J. Fish Biol..

[21] Zhang Z., Li J., Su C., Wang Z., Li Y., Li D., Chen Y., Liu C. (2024). A Method for Counting Fish Based on Improved YOLOv8. Aquac. Eng..

[22] Li D., Hao Y., Duan Y. (2020). Nonintrusive Methods for Biomass Estimation in Aquaculture with Emphasis on Fish: A Review. Rev. Aquac..

[23] Lomeli M.J.M., Wakefield W.W. (2019). The Effect of Artificial Illumination on Chinook Salmon Behavior and Their Escapement out of a Midwater Trawl Bycatch Reduction Device. Fish Res..

[24] Saberioon M.M., Cisar P. (2016). Automated Multiple Fish Tracking in Three-Dimension Using a Structured Light Sensor. Comput. Electron. Agric..

[25] Golovinskaya K. (1971). Breeds of Carps and Other Fishes.

[26] García-Vega A., Sanz-Ronda F.J., Fuentes-Pérez J.F. (2017). Seasonal and Daily Upstream Movements of Brown Trout *Salmo Trutta* in an Iberian Regulated River. Knowl. Manag. Aquat. Ecosyst..

[27] Quaranta E., Bejarano M.D., Comoglio C., Fuentes-Pérez J.F., Pérez-Díaz J.I., Sanz-Ronda F.J., Schletterer M., Szabo-Meszaros M., Tuhtan J.A. (2023). Digitalization and Real-Time Control to Mitigate Environmental Impacts along Rivers: Focus on Artificial Barriers, Hydropower Systems and European Priorities. Sci. Total Environ..

[28] Baumgartner L.J., Bettanin M., McPherson J., Jones M., Zampatti B., Beyer K. (2012). Influence of Turbidity and Passage Rate on the Efficiency of an Infrared Counter to Enumerate and Measure Riverine Fish. J. Appl. Ichthyol..

[29] Baumgartner L.J., Bettanin M., McPherson J., Jones M., Zampatti B., Beyer K. (2010). Assessment of an Infrared Fish Counter (Vaki Riverwatcher) to Quantify Fish Migrations in the Murray-Darling Basin. Fisheries Final Report Series N° 116.

[30] Shardlow T.F., Hyatt K.D. (2004). Assessment of the Counting Accuracy of the Vaki Infrared Counter on Chum Salmon. N. Am. J. Fish. Manag..

[31] Eatherley D.M.R., Thorley J.L., Stephen A.B., Simpson I., MacLean J.C., Youngson A.F. (2005). Trends in Atlantic Salmon: The Role of Automatic Fish Counter Data in Their Recording.

[32] Santos J.M., Pinheiro P.J., Ferreira M.T., Bochechas J. (2008). Monitoring Fish Passes Using Infrared Beaming: A Case Study in an Iberian River. J. Appl. Ichthyol..

